# Food for Song: Expression of C-Fos and ZENK in the Zebra Finch Song Nuclei during Food Aversion Learning

**DOI:** 10.1371/journal.pone.0021157

**Published:** 2011-06-10

**Authors:** Kirill Tokarev, Anna Tiunova, Constance Scharff, Konstantin Anokhin

**Affiliations:** 1 Department of the Neurobiology of Memory, P. K. Anokhin Institute of Normal Physiology, Russian Academy of Medical Sciences, Moscow, Russia; 2 Department of Animal Behavior, Freie Universität Berlin, Berlin, Germany; Rutgers University, United States of America

## Abstract

**Background:**

Specialized neural pathways, the song system, are required for acquiring, producing, and perceiving learned avian vocalizations. Birds that do not learn to produce their vocalizations lack telencephalic song system components. It is not known whether the song system forebrain regions are exclusively evolved for song or whether they also process information not related to song that might reflect their ‘evolutionary history’.

**Methodology/Principal Findings:**

To address this question we monitored the induction of two immediate-early genes (IEGs) c-Fos and ZENK in various regions of the song system in zebra finches (*Taeniopygia guttata*) in response to an aversive food learning paradigm; this involves the association of a food item with a noxious stimulus that affects the oropharyngeal-esophageal cavity and tongue, causing subsequent avoidance of that food item. The motor response results in beak and head movements but not vocalizations. IEGs have been extensively used to map neuro-molecular correlates of song motor production and auditory processing. As previously reported, neurons in two pallial vocal motor regions, HVC and RA, expressed IEGs after singing. Surprisingly, c-Fos was induced equivalently also after food aversion learning in the absence of singing. The density of c-Fos positive neurons was significantly higher than that of birds in control conditions. This was not the case in two other pallial song nuclei important for vocal plasticity, LMAN and Area X, although singing did induce IEGs in these structures, as reported previously.

**Conclusions/Significance:**

Our results are consistent with the possibility that some of the song nuclei may participate in non-vocal learning and the populations of neurons involved in the two tasks show partial overlap. These findings underscore the previously advanced notion that the specialized forebrain pre-motor nuclei controlling song evolved from circuits involved in behaviors related to feeding.

## Introduction

**Figure 1:**
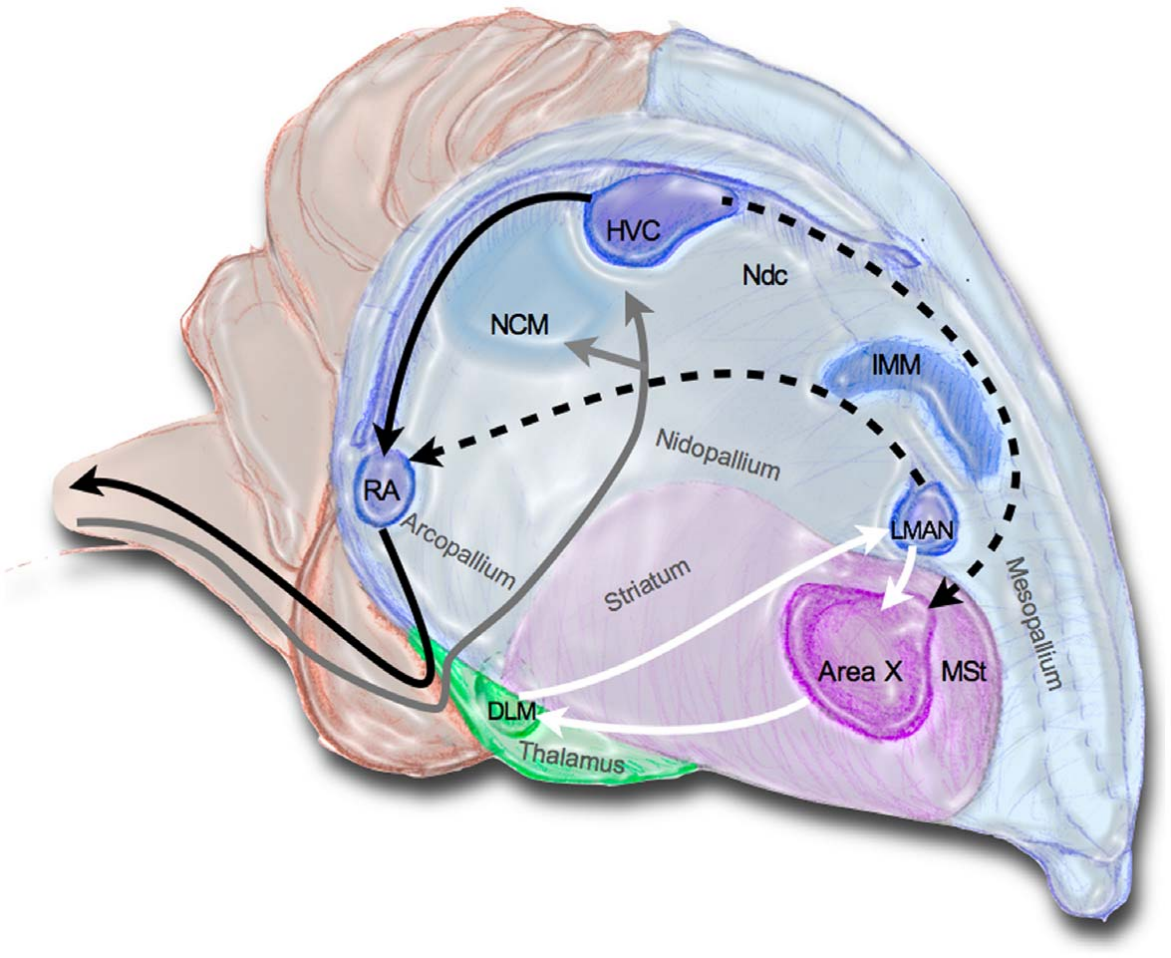
A schematic semi-3D view of a songbird brain depicting the brain regions relevant for this study. The structures of the song system are organized into three pathways: posterior (axonal connections shown in black), anterior (connections in white, and connections between anterior and posterior are dashed), and auditory (gray). Pallial regions are shaded in blue, basal ganglia in purple and thalamic in green. MSt, IMM and Ndc are regions involved in food aversion learning in chicken.

Spoken language, speech, is learned during early childhood via imitation. Besides humans, a small number of vertebrate taxa, among them songbirds, are capable of vocal learning. There are numerous analogies in the mechanisms of vocal learning in humans and songbirds [1], at the molecular [2], [3], neuroanatomical [4], [5] and behavioral levels [6], [7]. Songbirds, like humans, evolved specialized areas in the forebrain controlling and processing learned vocalizations, called the song system (**Figure 1**). These neural circuits distinguish them from the birds and mammals that lack vocal production learning. Vocalizations in birds that do not learn their song and non-learned vocalizations in songbirds are controlled by subtelencephalic circuits [8]–[10], and mammals that use ‘innate’ vocalizations, e.g. not learned imitatively, can do so in the absence of cortical control [11]. Vocal production learning may have evolved in several vertebrate taxa independently [4]. Independent evolution has also been suggested for electric communication systems and their underlying neural networks in two different groups of weakly electric fishes, African *Mormyridae* and South American *Gymnotiformes* [12], and for similar neural mechanism that allows precise temporal coding in the auditory systems of crocodilians, birds and mammals [13], [14]. Alternatively, vocal learning might have been present in common ancestors but was lost in many descendent groups [4]. How the brain regions required for learned speech in humans and learned song in songbirds evolved from species that lacked these traits is not known, although it has been proposed that they duplicated from adjacent motor areas [4], [15], [16] or arose from auditory regions [17], [18]. It is also not known whether the song nuclei are specialized exclusively for vocal behavior or whether they are implicated in other types of behavior as well. The latter might reflect their ‘evolutionary history’.

The neural substrates for non-vocal behaviors such as spatial learning [19]–[21] and sexual imprinting [22], [23] in zebra finches lie outside the song system. For instance, the zebra finch hippocampus is involved in spatial learning but it is not necessary for vocal behavior and its development, despite evidence that it may be involved in some aspects of song perception [24], [25]. Likewise, locomotor behavior such as hopping induces immediate-early gene (IEG) activity in many regions surrounding song nuclei, but not within the song nuclei themselves [16].

To test the hypothesis that song nuclei are not limited to processing song behavior in the present work we used one-trial food aversion learning in zebra finches, in which a single exposure to a novel food was associated with an aversive taste and henceforth avoided. This type of learning has been well studied in young domestic chickens (*Gallus gallus domesticus)* [26], and we chose it as a behavior paradigm that, like singing, involves head, beak, tongue and oropharyngeal-esophageal cavity movements, but does not involve song perception or song production itself. We therefore evaluated the pattern of induction of two IEGs c-Fos and ZENK, in zebra finches in regions of the song system after food aversion learning and after song production and/or perception, because the latter is well documented to occur in songbirds [27]–[30].

Structures involved in passive avoidance learning in juvenile domestic chickens were first identified by the analysis of metabolic activity in the brain [31] and confirmed by lesions and pharmacological intervention studies [32], [33]. This training induces a cascade of intracellular events, resulting in IEG (c-Fos) expression in intermediate medial mesopallium (IMM) and medial striatum (MSt) [34] (for review see [26]). Dorsocaudal nidopallium (Ndc) and dorsal nidopallium (Nd) are also involved in passive avoidance learning in 15 day-old chickens (unpublished results). Intriguingly, the song nucleus HVC (used as a proper name) is argued to be paralogous to Nd of chickens [35].

We used expression of two transcription factors c-Fos and ZENK (a.k.a. Zif268, Egr1, NGFI-A, Krox-24), to visualize neural activity in different brain regions [36]–[39]. C-Fos expression is required for long-term memory formation in passive avoidance training in 1 day-old domestic chickens, evidenced by amnesic effects after intracranial administration of antisense oligonucleotides [40]. In addition, inhibition of the extracellular signal-regulated kinase signaling pathway, normally leading to ZENK expression, leads to poor song learning success in zebra finches [41]. In songbirds, both c-Fos expression and ZENK have been used previously to map brain regions activated as a result of singing, hearing song, locomotion and socio-sexual behaviors [16], [27]–[30], [42], [43] and are known to regulate expression of different late genes such as synapsins, involved in neuronal plasticity [44]. Singing as a motor act, not requiring auditory feedback, induces c-Fos and ZENK expression in HVC and RA (robust nucleus of arcopallium) [16], [28]–[30], as well as in Area X and LMAN (lateral magnocellular nucleus of anterior nidopallium) when the bird does not address the song to another individual [16], [29]. In contrast, hearing song is accompanied by c-Fos and ZENK expression in several auditory regions including medial nidopallium (NCM) and areas immediately adjacent to HVC and RA (called shelf and cup, respectively) but not in these latter nuclei themselves [16], [27]. Body movement like hopping and walking activates expression of these genes in the areas around song nuclei but not in HVC shelf and RA cup [16]. In songbird brains, c-Fos and ZENK can be induced in the same neurons by the same stimuli [45] or the same stimuli can cause differential expression. For instance, in juvenile zebra finches c-Fos is induced in caudal NCM after exposure to conspecific songs in females, while ZENK is expressed only in males [24]. However, in adulthood, males and females show similar patterns of activation in NCM in response to conspecific song [45]. Although ZENK and c-Fos may show different activation patterns, they have been rarely studied in the same brain tissue in songbirds. Using triple fluorescent immunocytochemistry, we explored their neuroanatomical and cellular colocalization in different behavioral conditions. In summary, we used the transcription factors c-Fos and ZENK (1) to analyze the possible involvement of song nuclei in one-trial food aversion learning, a non-vocal behavior, and (2) to study the overlap of expression patterns of ZENK and c-Fos in the zebra finch forebrain. Our data show that two song system motor regions, HVC and RA, are activated after a one-trial food aversion learning task in the absence of singing or hearing song. The data also allow us to compare brain structures activated during one-trial aversion learning in vocal learning songbirds with those of non vocal learning domestic chickens (based on previous works and our unpublished data).

## Results

### Behavior

In the present work we developed a new one-trial learning task for adult songbird, resulting in avoidance of a novel, aversive food. Similarly to the traditional passive avoidance learning model in domestic chicken hatchlings [26], training in this task was based on presenting of a novel potential food object, parsley, covered with the aversive methylanthranilate (MeA), which is used as a bird repellent for seed crops [46].

During the food aversion experiment the latency to peck at the parsley was less than 2 min in all tested birds. All birds (n = 10) pecked the parsley once and displayed an aversive reaction similar to that characteristic for chicken hatchlings (head shaking and beak wiping [47]). Both pecking and aversive reaction involved moving the beak and head (and probably tongue as well), which is also the case during singing, but the birds of the food aversion group did not sing during the experiment. During the rest of the 5 min session the birds kept flying around the cage agitatedly. However, flying does not induce IEG expression in the song nuclei [16]. After the parsley was removed, the birds' behavior returned to pre-test levels of activity (data not shown). In the test administered 90 min after the training, the zebra finches were presented simultaneously with parsley and dill, both not treated with MeA. None of the ten birds touched the parsley, while eight tried the dill leaves.

To control for spontaneous preferences for parsley or dill, a different set of 21 adult zebra finches that had not been exposed to either dill or parsley previously, were presented with non-coated parsley or dill: 5 pecked at dill, 5 at parsley, 5 consumed both greens and 6 touched neither of them during a 3 min preference test. Thus in adult male zebra finches, a single association of a novel food object with aversive taste produced selective avoidance of this object at a subsequent test. This passive avoidance training had been previously shown in domestic chicken hatchlings but not in songbirds [26].

To be able to assess the specificity of the possible induction of IEGs in different parts of the song system as a result of food aversion training we designed 3 control groups, consisting of 6 animals each. Birds in the ‘song control group’ heard tape recorded song (30 presentations during the first 15 min of a 90 min experimental session) and also sang themselves. The number of song bouts (succession of uninterrupted song motifs) per bird averaged 111, varying from 19 to 224. Singing activates IEGs in many song control regions as well as auditory regions (as singing implies also hearing one's self), whereas mere hearing song induces IEGs in auditory regions but not in regions controlling singing [16]. A second control group, the ‘active control’, was exposed to identical conditions except for the noxious stimulus. Birds in this group performed their regular daily activity including active eating, flying and calling but did not sing nor did they hear song. The birds in both food aversion and active control groups were housed in neighboring cages with visual contact, therefore reducing the possibility that they would produce a confounding type of vocalization, long calls. Long calls contain a learned component and are produced by zebra finches in visual isolation [48]. The third control group, the ‘quiet control’ served to establish basal levels of IEG expression after they had slept for 8 hours overnight in a quiet room.

### Immunohistochemistry

To compare expression patterns of c-Fos and ZENK in the 4 behavioral conditions we chose 8 telencephalic structures for analysis: 2 nuclei of the posterior pathway of the song system (HVC and RA), 2 of the anterior forebrain pathway (Area X and LMAN), a secondary auditory area specialized for the perception of conspecific vocalizations (NCM), and 3 areas involved in food aversion learning in young chickens (IMM, MSt and Ndc). Their location is schematically depicted in **Figure 1**. In all 8 examined brain structures expression of both transcription factors was lowest in the quiet control group. This level was considered to be the baseline expression. Since the paired-samples t-test revealed no significant differences between left and right hemispheres, the data from both hemispheres were averaged.

#### IEGs expression in the song nuclei

**Figure 2:**
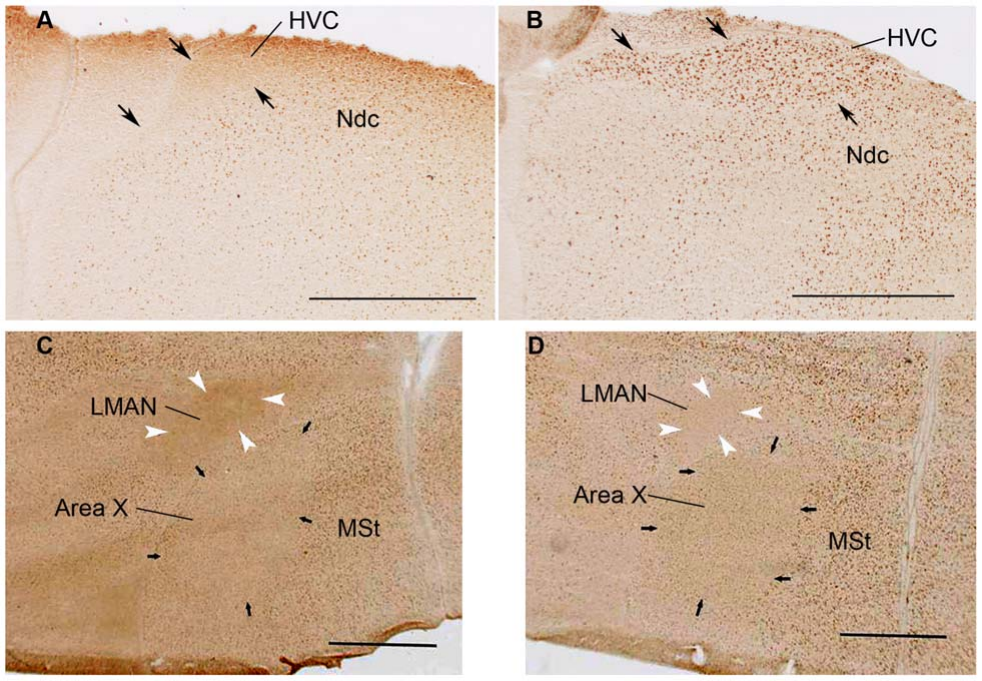
Expression of ZENK and c-Fos in HVC, Area X and LMAN after food aversion learning. Expression of ZENK protein (A,C) and c-Fos protein (B,D) was revealed by DAB immunohistochemistry (brown nuclear staining). In HVC, ZENK was not expressed following the food aversion paradigm (***A,*** HVC delineated by black arrows), whereas c-Fos showed robust expression (***B***). LMAN (delineated by white arrowheads) and Area X (delineated by black arrows) in non-singing conditions expressed neither ZENK (***C***) nor c-Fos (***D***). Transverse sections. Scale bar, 1 mm.

**Figure 3:**
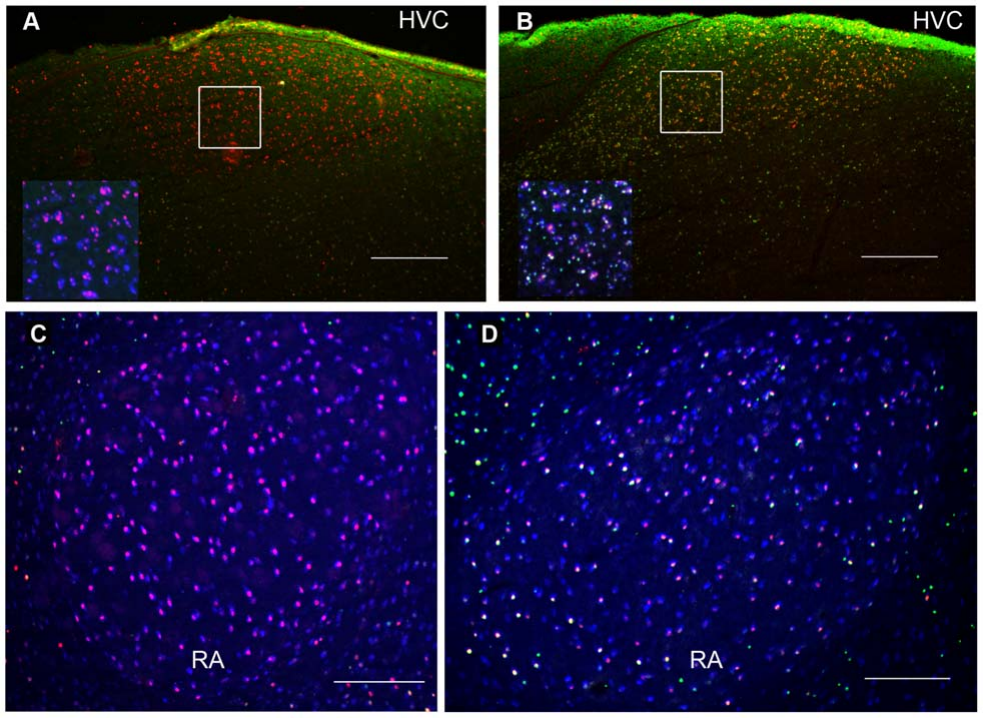
Fluorescent immunostaining of IEG expression in HVC and RA after food aversion learning (*A,C*) or after singing (*B,D*). C-Fos (red nuclear label) was expressed in HVC and RA after both behaviors, whereas ZENK (green nuclear label) only in the song group (colocalized expression of both IEGs, yellow, ***B***, or white in the insert of ***B,*** and in ***D***); neuron-specific NeuN labeling is shown in blue cytoplasmatic and nuclear label. Transverse sections. Scale bars, 0.2 mm (***A*** and ***B***), 0.1 mm (***C*** and ***D***).

**Figure 4:**
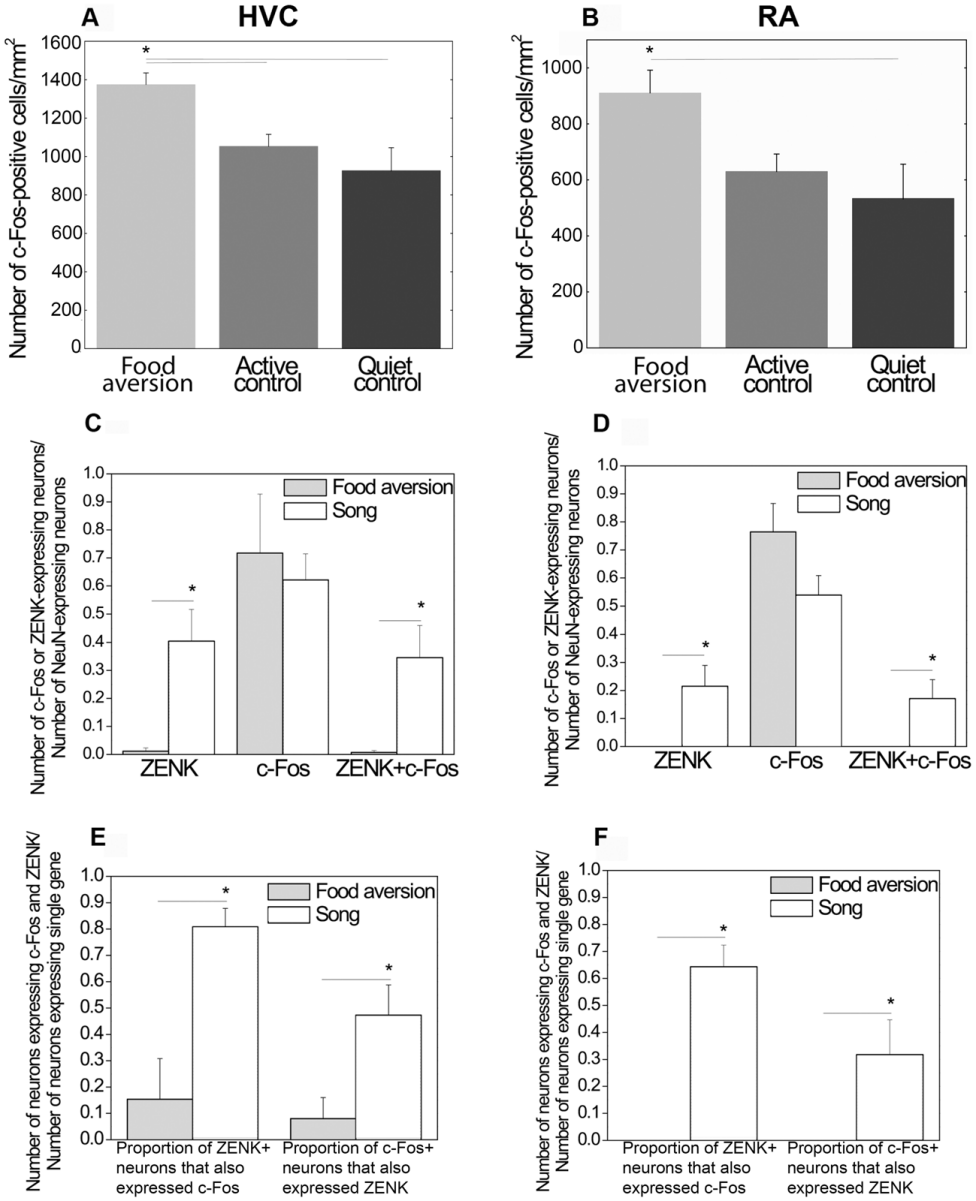
Quantification of IEG expression in HVC (*A,C,E*) and RA (*B,D,F*) in different experimental conditions. ***A***. Density of c-Fos-positive cells in HVC in the food aversion group compared to control groups. * = p<0.05, one-way ANOVA (F = 7.85; df1 = 2; df2 = 12) followed by LSD post-hoc test. ***B***. Density of c-Fos-positive cells in the food aversion group compared to control groups. * = p<0.05, one-way ANOVA (F = 4.14; df1 = 2; df2 = 12) followed by LSD post-hoc test. ***C***. C-Fos and ZENK expression in the food aversion group and song control group. Proportions of mature (NeuN-expressing) HVC neurons expressing ZENK or/and c-Fos are shown. * = p<0.05, independent-samples t-test. ***D***. C-Fos and ZENK expression in the food aversion group and song control group. Proportions of mature (NeuN-expressing) RA neurons expressing ZENK or/and c-Fos are shown. * = p<0.05, independent-samples t-test. ***E***. C-Fos and ZENK expression in the food aversion group and song control group. Number of HVC neurons expressing both genes is shown as proportions of the numbers of ZENK- or c-Fos-expressing neurons. * = p<0.05, independent-samples t-test. ***F***. C-Fos and ZENK expression in the food aversion group and song control group. Number of RA neurons expressing both genes is shown as proportions of the numbers of ZENK- or c-Fos-expressing neurons. * = p<0.05, independent-samples t-test. Error bars, S.E.M.

After one trial food aversion training ZENK expression was not detected in HVC and RA but c-Fos was (**Figure 2 A,B**). In Area X and LMAN, neither of the IEGs were expressed after the aversive food training (**Figure 2 C,D**). We compared this novel finding to the well known IEG induction in HVC and RA as a result of singing (**Figure 3**). C-Fos was induced in HVC and RA to a similar degree by singing (in the song control group) as by food aversion training, although those birds did not sing (**Figure 3**). In contrast, ZENK induction was reliably detected in HVC and RA only as a consequence of singing (**Figure 3B,D**), but not in response to food aversion (**Figure 3 A,C**). Quantitative analysis of these results revealed that in HVC there were significantly more c-Fos positive neurons after the aversive food training than there were in the active and quiet controls (ANOVA, df 14 F = 7.85, p = 0.007; post-hoc LSD test food aversion vs active control p = 0.022, food aversion vs quiet control p = 0.003; **Table 1**; **Figure 4A**). In RA, there was also a significant difference between the three groups (ANOVA, df 14 F = 4.14, p = 0.043, **Figure 4B**). Post-hoc tests showed that significantly more neurons expressed IEGs after food aversion than in the quiet control (p = 0.017), but for the comparison between food aversion and active control group there was only a trend (post-hoc LSD test, p = 0.078). To assess the magnitude of induction due to the food aversion paradigm, we then compared the proportions of IEG expressing neurons to those induced by singing. In HVC and RA, food aversion induced the same amount of c-Fos as did singing (**Figure 4C,D**). Because roughly two thirds of HVC and RA neurons expressed c-Fos under both conditions (t-test, p = 0.394; **Figure 4C,D**), some HVC and RA neurons must express c-Fos both as a result of singing as well as a result of the food aversion paradigm in the absence of singing. Many double-labeled neurons positive for both c-Fos and ZENK were found after singing but hardly any (in HVC) or none (in RA) after food aversion (Fig 4C,D). The proportion of c-Fos expressing neurons that also expressed ZENK in the song group ranged from 45% in HVC to 35% in RA (**Fig 4A,B**).

**Table 1:** Densities of IEG positive cells in the analyzed structures in the food aversion task in comparison to active and quiet controls.

	Food aversion		Active control		Quiet control	
	ZENK	c-Fos	ZENK	c-Fos	ZENK	c-Fos
HVC	≈0	1374±65	≈0	1052±66	≈0	926±126
RA	≈0	910±86	≈0	629±66	≈0	523±129
Area X	≈0	≈0	≈0	≈0	≈0	≈0
LMAN	≈0	≈0	≈0	≈0	≈0	≈0
NCM	499±120	502±112	330±50	415±62	97±71	128±33
Ndc	704±138	521±82	639±133	442±54	97±71	148±42
IMMrostral	1533±134	1617±64	1429±130	1548±76	972±106	1403±135
IMMcaudal	828±82	1091±96	730±88	1063±34	126±44	576±43
MSt	1328±82	1285±140	965±75	1187±131	508±168	609±54

(Means ± SE, per mm^2^).

Abbreviations: HVC – nucleus HVC of the nidopallium, formerly, the high vocal center; RA – robust nucleus of the arcopallium; Area X – Area X of the medial striatum; LMAN – lateral magnocellular nucleus of the anterior nidopallium; NCM – caudomedial nidopallium; Ndc – dorsocaudal nidopallium; IMM – intermediate medial mesopallium; MSt – medial striatum.

**Figure 5:**
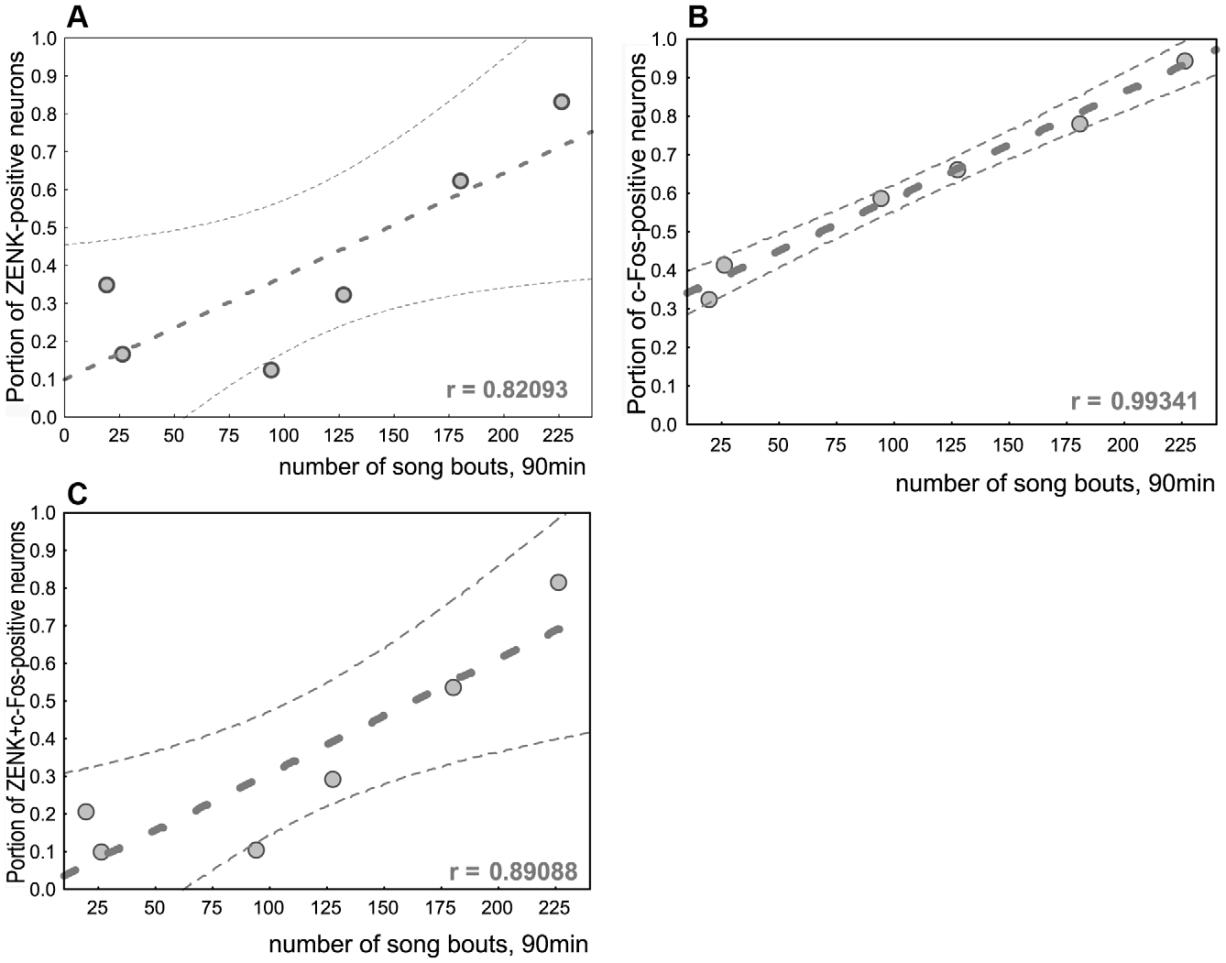
Relationship between the amount of singing and the number of HVC neurons expressing IEGs. Correlation of the proportions of HVC neurons, expressing ZENK (***A***), c-Fos (***B***) or both genes (***C***) (y axis), with the number of song bouts (x axis). Dashed lines, 95% confidence interval; r, linear correlation coefficient.

In accordance with previous findings [29], [30], we found a significant positive linear correlation between the number of song bouts produced and the proportions of HVC neurons expressing either one or both of the studied IEGs (Figure 5).

Could the c-Fos expression be related to vocalizations in the food aversion group, even though they did not sing? Attempts to sing do not produce ZENK induction in HVC or RA [28], and we also did not find any ZENK induction in the food aversion group (**Figure 2A**, **Figure 3A,C**, **Figure 4C,D**), even though 2 birds attempted to sing and had to be interrupted by a hand motion from a nearby observer (see methods). It is not known whether c-Fos can be induced by singing attempts alone, but if so, this would have equally affected birds in the active control group where 3 birds attempted to sing as well. Yet, c-Fos induction in the food aversion group was significantly higher in HVC than the expression in the active control group. Furthermore, there was no significant difference between the quiet control group, where attempts to sing did not occur, and the active control group, where 3 birds did attempt to sing. Taken together, the likelihood that the c-Fos induction in the food aversion group stemmed from attempts to sing is remote. Another source of c-Fos induction could be distance calls. They contain a learned component and require an intact HVC and RA [48], and therefore it is possible that they would induce c-Fos in these nuclei. We did not quantify distance calling, but because distance calls are mostly produced by birds that can hear but not see each other [9], [10], it is unlikely that substantial amounts of distance calling occurred in the active control and food aversion group that were caged beside each other allowing visual contact. Whatever calling did occur would be expected to have been similar in both groups. Therefore distance calls are also unlikely to account for the observed much stronger c-Fos induction in HVC of the food aversion than the active control group.

**Figure 6:**
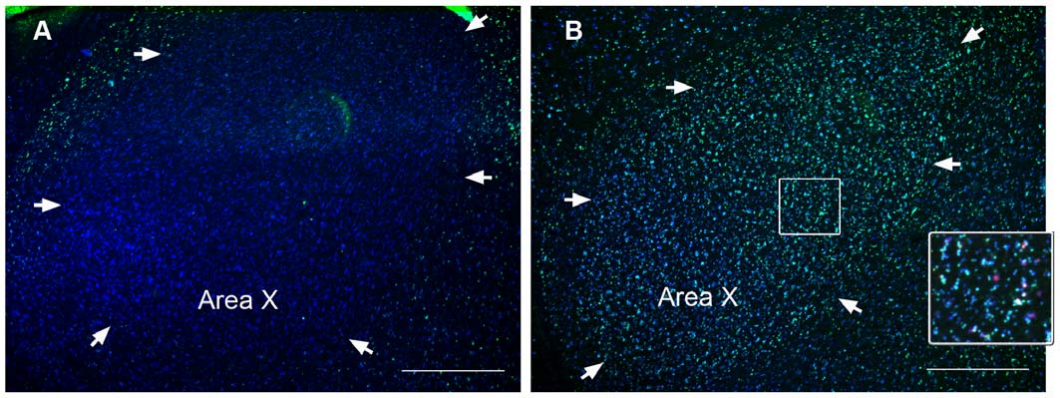
Fluorescent immunostaining of IEG expression in Area X after food aversion learning (*A*) or singing (*B*). ZENK expression (green nuclear stain) in Area X neurons (labeled by NeuN, blue cytoplasmatic and nuclear stain) occurred only in the song control group (***B***), as did the expression of c-Fos (red nuclear stain, ***B, insert***).Transverse sections. Scale bar, 0.5 mm.

Food aversion training and regular feeding behavior of the active control birds induced hardly any expression of ZENK or c-Fos in Area X and LMAN of the telencephalic anterior forebrain pathway (AFP) (**Figure 2C,D** and **Figure 6A**). In contrast, undirected singing induced ZENK and c-Fos expression in many neurons of these AFP nuclei, as reported previously [16], [29], [49] (**Figure 6B**).

**Figure 7:**
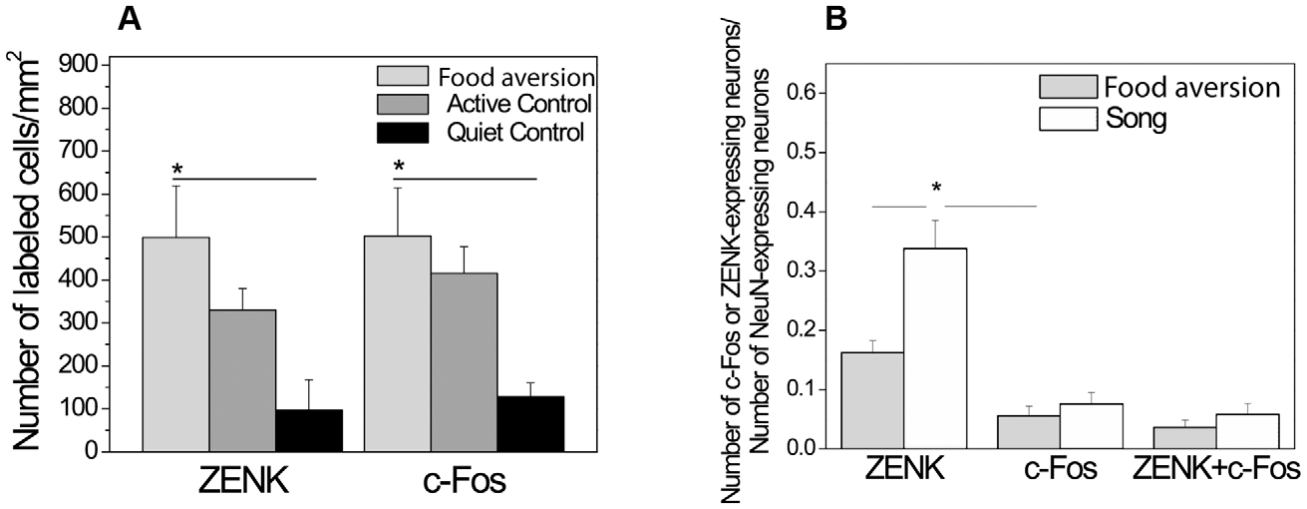
Quantification of IEG expression in NCM under different experimental conditions. ***A***. Density of ZENK- and c-Fos-positive cells in the food aversion group compared to control groups. * = p<0.05, one-way ANOVA (F = 4.29 for ZENK, F = 4.19 for c-Fos; df1 = 2; df2 = 10) followed by LSD post-hoc test. ***B***. C-Fos and ZENK expression in the food aversion group or song control group. Proportions of mature (NeuN-expressing) NCM neurons expressing ZENK or/and c-Fos are shown. * = p<0.05, independent-samples t-test. Error bars, S.E.M.

**Figure 8:**
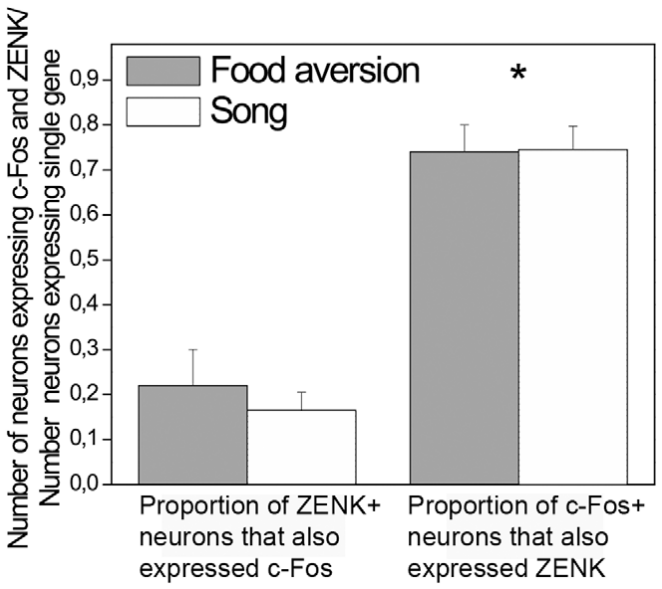
C-Fos and ZENK expression in NCM neurons in the food aversion group and song group. Number of NCM neurons, expressing both genes, are shown as proportions of the numbers of ZENK- or c-Fos-expressing neurons. With both behaviors about three quarters of c-Fos-positive NCM neurons also expressed ZENK, whereas among ZENK-positive neurons the proportion of those containing also c-Fos was significantly lower. * = p<0.05, independent-samples t-test. Error bars, S.E.M.

C-Fos and ZENK were also expressed in the auditory region NCM of the birds in all groups, to varying degrees, (**Figure 7A**), presumably due to different levels of auditory stimulation. ZENK was expressed at substantially higher level in the song control group than the food aversion group (t-test, p = 0.039; **Figure 7B**). The density of c-Fos and ZENK labeled cells in the NCM of the food aversion group was significantly higher than in the quiet control group (ANOVA, df 14, F = 4.186 and F = 4.291, p = 0.048 and p = 0.039, respectively; post-hoc LSD test, p = 0.017 and p = 0.013, respectively). Since there was no significant difference between the active control and the food aversion learning group (post-hoc LSD test, p = 0.416 for c-Fos and p = 0.215 for ZENK, **Figure 7A**), the IEG expression in the latter two groups was likely due to hearing calls [50]–[52] and wing beats [16]. C-Fos expression was observed mostly in the same neurons that expressed ZENK (**Figure 8**).

#### IEGs expression in songbird regions homologous to those involved in passive avoidance learning in domestic chickens

**Figure 9:**
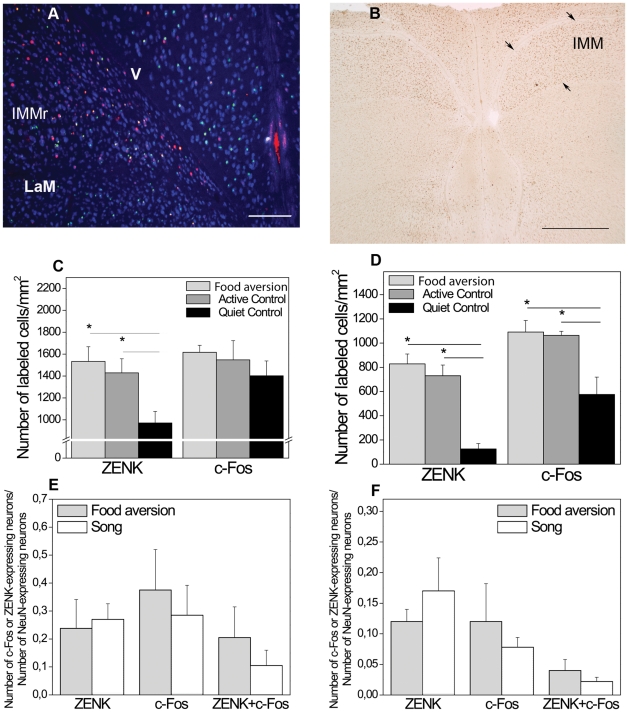
Visualization and quantification of IEG expression in IMM in different experimental conditions. ***A***. A representative image of the rostral part of IMM (between the ventricle, V, and mesopallial lamina, LaM) with ZENK (green) and c-Fos (red) expressing neurons (NeuN, blue) in the food aversion group. Transverse section. Scale bar, 0.1 mm. ***B*** *.* A representative image of the caudal part of IMM with DAB-labeled (brown) c-Fos staining after the food aversion task (black arrows indicate the IMM boundaries in the right hemisphere). Transverse section. Scale bar, 1 mm. ***C*** *.* Density of ZENK- and c-Fos-positive cells in the rostral part of IMM in the food aversion group compared to control groups.* = p<0.05, one-way ANOVA (F = 5.56 for ZENK, F = 0.75 for c-Fos; df1 = 2; df2 = 13) followed by LSD post-hoc test. ***D*** *.* Density of ZENK- and c-Fos-positive cells in the caudal part of IMM in the food aversion group compared to control groups.* = p<0.05, one-way ANOVA (F = 24.89 for ZENK, F = 8.01 for c-Fos; df1 = 2; df2 = 13) followed by LSD post-hoc test. ***E and F***. Proportions of mature (NeuN-expressing) neurons expressing ZENK or/and c-Fos are shown for the rostral (***E***) and caudal (***F***) parts of IMM. Error bars, S.E.M.

The IMM region is present at two levels, “rostral” and “caudal” (IMHV on planes A2.6 and A1.6, respectively, in the canary stereotaxic atlas, [53]; a medial region between the ventricle and mesopallial lamina, see **Figure 9A,B** for representative images). In the rostral IMM, high densities of c-Fos-immunoreactive cells were observed in the quiet control, active control and food aversion groups alike (**Table 1; C**). In contrast, in the caudal part of IMM significantly more cells expressed c-Fos in the active control and food aversion groups than in quiet controls (ANOVA, df 15, F = 8.006, p = 0.005) (**Table 1**; **Figure 9D**). ZENK induction in these two groups compared to the quiet control was observed in both rostral and caudal regions of IMM (ANOVA, df 15, F = 5.555 and F = 24.887, p = 0.018 and p = 0.000, respectively; **Table 1**; **Figure 9C,D**). In the song control group, portions of neurons expressing c-Fos and/or ZENK were comparable to those in the food aversion group (**Figure 9E,F**).

**Figure 10:**
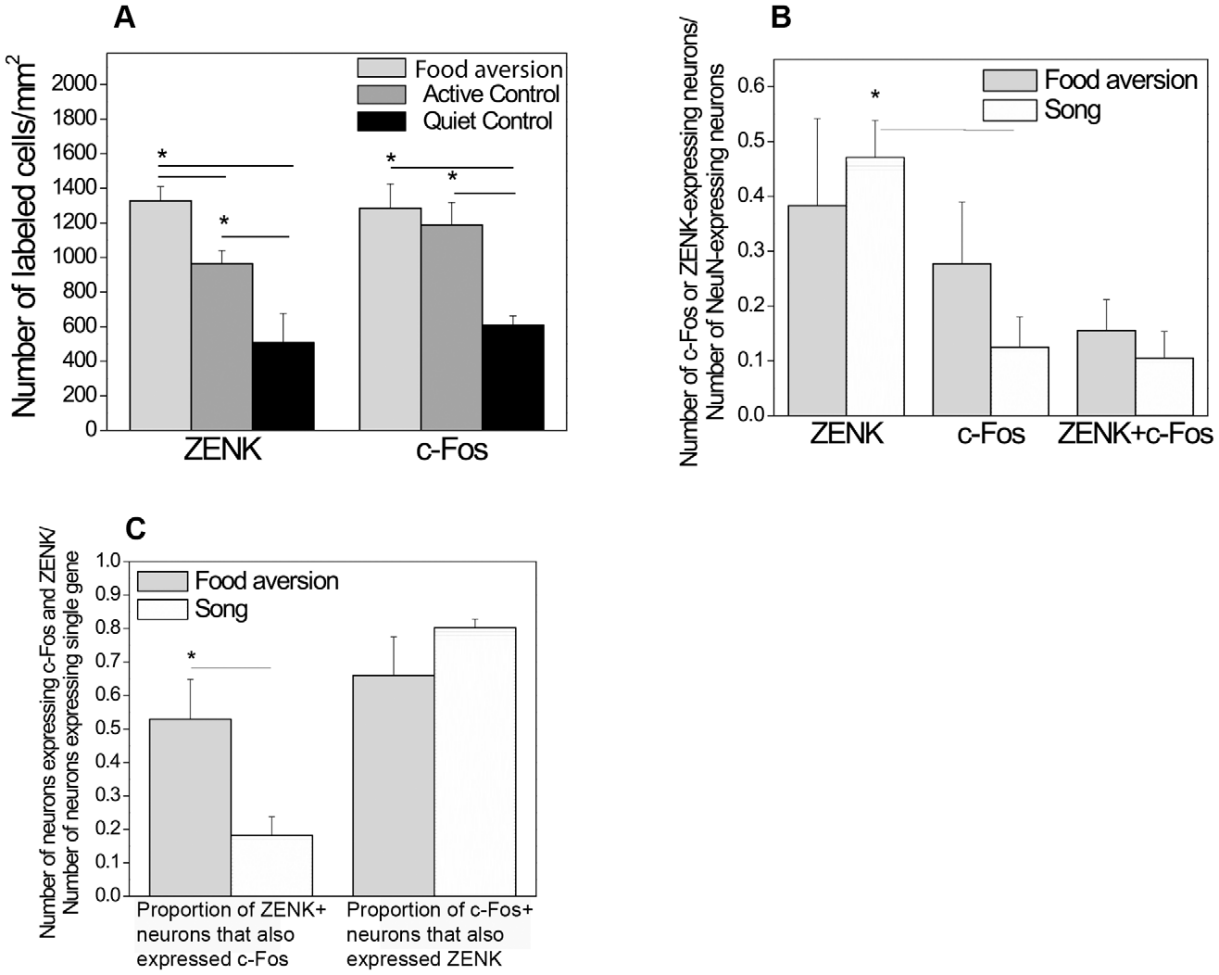
Quantification of IEG expression in MSt in different experimental conditions. ***A***. Density of ZENK- and c-Fos-positive cells in the food aversion group compared to control groups. * = p<0.05, one-way ANOVA (F = 13.52 for ZENK, F = 9.18 for c-Fos; df1 = 2; df2 = 13) followed by LSD post-hoc test. ***B***. C-Fos and ZENK expression in the food aversion group and song control group. Proportions of mature (NeuN-expressing) MSt neurons expressing ZENK or/and c-Fos are shown. ***C***. C-Fos and ZENK expression in the food aversion group and song control group. Number of MSt neurons expressing both genes is shown as proportions of the numbers of ZENK- or c-Fos-expressing neurons. * = p<0.05, independent-samples t-test. Error bars, S.E.M.

Since Area X lies in the dorsolateral part of MSt, and only the birds of the song control group had IEG expression in Area X (see above, **Figure 6B**), we assessed IEG expression in the medial part of MSt (**Figure 2C,D**). This region seems to partially overlap with the anterior striatum, which was identified as a movement related area in a previous study [16]. In the MSt, both c-Fos and ZENK expression was enhanced in the food aversion and active control groups compared to the quiet control; moreover, the number of ZENK expressing neurons was significantly higher in the food aversion group than in the active control (ANOVA, df 15, F = 9.18 and F = 14.523, p = 0.003 and p = 0.001, respectively) (**Table 1**; **Figure 10A**). In the song control group, contrary to the food aversion group, fewer neurons expressed c-Fos than ZENK (15% and 50% respectively) (t-test, p = 0.043) (**Figure 10B**). Even though the song and food aversion group did not differ in the proportions of neurons expressing ZENK (**Figure 10B**), they did differ in the proportions of those ZENK-immunoreactive neurons that also expressed c-Fos (t-test, p = 0.044; **Figure 10C**).

**Figure 11:**
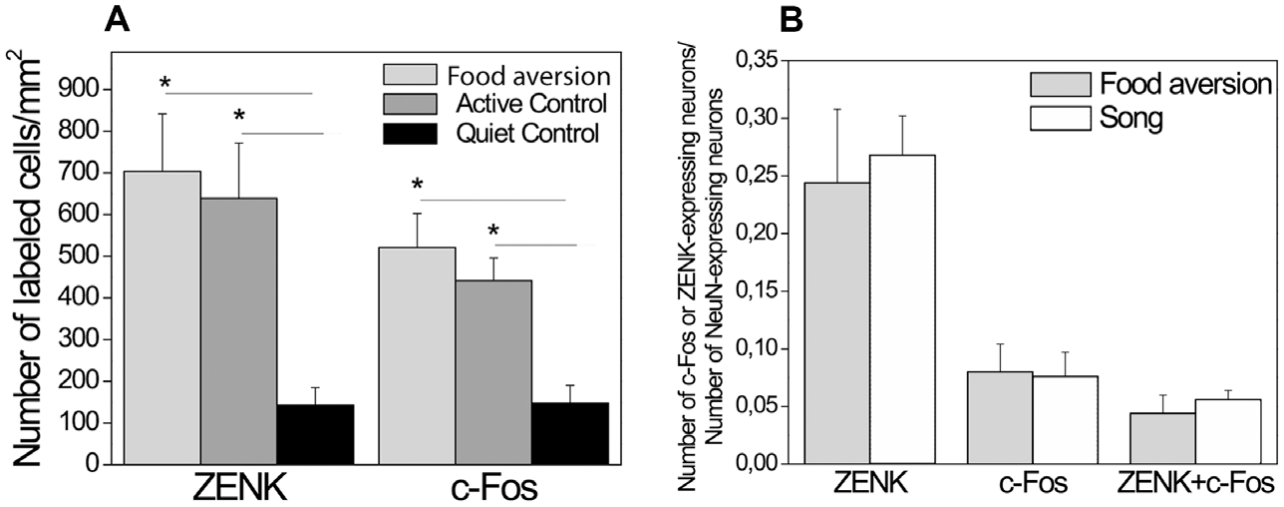
Quantification of IEG expression in Ndc in different experimental conditions. ***A***. Density of ZENK- and c-Fos-positive cells in the food aversion group compared to control groups. * = p<0.05, one-way ANOVA (F = 6.7 for ZENK, F = 8.96 for c-Fos; df1 = 2; df2 = 13) followed by LSD post-hoc test. Error bars, S.E.M. ***B***. C-Fos and ZENK expression in the Ndc neurons in the food aversion group and song group. Proportions of mature (NeuN-expressing) neurons expressing ZENK or/and c-Fos are shown. Error bars, S.E.M.

Levels of both c-Fos and ZENK in Ndc (its location is shown on **Figure 2**) were significantly elevated in the food aversion and active control groups (ANOVA, df 15, F = 8.958 and F = 6.7, p = 0.004 and p = 0.01, respectively) (**Figure 11A**), but the proportions of Ndc neurons expressing either ZENK or c-Fos or both simultaneously did not differ significantly between the food aversion and song control groups (**Figure 11B**).

## Discussion

### Comparison of regions of the song system involved in vocal and non-vocal learning

Although the song system of zebra finches was studied in numerous experiments in the context of vocal learning, song production and song processing (for reviews see [54]), the present work examined for the first time a possible involvement of the song nuclei in a learning task which does not implicate song production or perception. We analyzed the patterns and the magnitude of neural activity - as measured by IEG expression - associated with vocal and non-vocal behaviors. IEG induction after a non-vocal one trial aversive food learning paradigm was compared to the well known IEG induction as a result of singing in song motor regions, or as a result of hearing song in auditory song regions. Surprisingly, c-Fos was expressed in similarly high proportions of neurons after food aversion training and after singing in nuclei HVC and RA of the posterior motor pathway of the song system. In HVC, we found significantly more neurons expressing c-Fos after the aversive food training session than after active, non-aversive eating, and relative to the quiet control; similar trend was also in the nucleus RA but the difference reached significant level only compared to the quiet control. This was not the case in nuclei of the anterior forebrain pathway. Our results are thus consistent with heterogeneity of the song system structures with respect to their involvement in behavior unrelated to species-specific vocalization/hearing.

The fact that significantly more cells expressed c-Fos in the birds of the food aversion learning group than in the active control group indicates that in certain populations of HVC neurons c-Fos expression was associated with some aspect of the food aversion learning paradigm. Further studies are required to find out whether the neurons showing such activation in food aversion learning are distinct from those involved in song behavior. Further studies are also necessary to address which aspect of the food aversion paradigm results in the elevated c-Fos expression in HVC and RA.

Thus, in addition to singing, c-Fos expression can also be activated in HVC and RA during a food discrimination learning paradigm in the absence of singing. Basal expression of c-Fos in RA and HVC observed in control birds could be due to so far unknown physiological processes not related directly to singing as well. RA projects to a series of nuclei in the ventrolateral rhombencephalon linking vocal and respiratory control pathways [55]; these include respiratory premotor nuclei retroambigualis (Ram [56]) and parambigualis (Pam) and laryngeal motor neurons [57]. And even syringeal muscles themselves were recorded to be active during the expiratory phase of respiration [58]. Thus, it is possible that some neurons of the posterior pathway of the song system are activated during many types of regular behavior or even sleep, when the control of respiration is necessary, or when there is spontaneous bursting activity reminiscent of that during singing [59]. Respiration also needs to be modulated during feeding[60], [61], which could explain c-Fos expression in HVC and RA of the birds from the food aversion and active control groups as well.

Another explanation for the activation of HVC and RA in the absence of singing could also arise from head, beak, tongue and oropharyngeal-esophageal cavity movements related to feeding. IEG expression surrounding song nuclei, but not within them, occurs as a consequence of stereotypic body movements [16]. However, Feenders et al. did not analyze movements of the head, beak, or expanding the oropharyngeal-esophageal cavity, and given that those are closely tied to movements also occurring during singing [62], [63], they might be linked to c-Fos induction within HVC or RA in our study. Although there is apparently no direct projection from RA to jaw and lingual motor neurons, some data suggest the possibility of indirect connections between them [57], and comparative analysis points to a resemblance between the vocalization system in the budgerigar and the ‘feeding circuit’ of the mallard [64]. Thus, if the song system evolved as a specialization of preexisting motor pathways, it seems plausible that during some movements like hopping or wing whirring only areas adjacent to the song nuclei are activated [16], whereas during some behavior patterns including beak, tongue and oropharyngeal-esophageal cavity movements the song system may be activated as well (but as our data suggest, only c-Fos and only in its posterior pathway). Intriguingly, a motor origin of vocal learning has been proposed for human speech as well, and it was shown that spoken language and arm gestures are controlled by overlapping motor control systems [65]–[68]. These findings led to the suggestion that brain regions controlling speech production evolved from related areas of our primate ancestors, because in humans and macaques, respectively, these areas contain ‘mirror neurons’ that activate during both grasps movements and observing grasps (for reviews see [67], [69]). Furthermore, among arm movements interrelated with speech production were grasps of different food objects [66], which resembles the situation with birds in our experiments when they pecked the food and we observed transcriptional activation in the song system. HVC of zebra finches also contains mirror neurons involved in vocal production/perception [70], but whether a similar mechanism is also used for manipulating (food) objects with the beak remains to be elucidated.

Our observation that there were more c-Fos–positive cells in HVC after the food aversion task than after active feeding may reflect the possibility that associative learning of the bitterness and the novel food object was mediated by c-Fos in these nuclei. In fact, during vocal learning, HVC is required for learned song production, whereas unlearned song can be produced without it, under control of the AFP [71]. However, it is unlikely that HVC was involved in the sensory aspect of food aversion learning, as IEGs are not induced in this area while hearing song [28], [30], which would correspond to listening to the tutor as a part of vocal learning.

In NCM, high expression of c-Fos and ZENK was observed not only in the song control group but also in the food aversion learning and active control groups. NCM is considered to be an auditory area specialized for species-specific song perception [27], [36], [72], [73]. Conspecific calls can induce ZENK expression in NCM [50]–[52]. Therefore one likely explanation of enhanced ZENK expression in the food aversion and active control groups is due to the auditory stimuli heard by birds, most probably calls of other individuals, but perhaps also sounds caused by wing beats and hops [16]. ZENK expression in NCM of the song control group birds was twice higher than in the birds of the food aversion group. This suggests that even though certain transcriptional activation occurred in NCM during behavior unrelated to song, presentation of conspecific song produced changes in many more neurons.

We did not observe any induction of ZENK and c-Fos in Area X and LMAN during regular bird activity, including routine feeding behavior. Area X and LMAN were also unresponsive during the food aversion training. However, both transcription factors were detected in the surrounding regions (anterior nidopallium around LMAN and medial striatum around Area X) in all experimental groups. Thus, comparison of the expression patterns produced by vocal and non-vocal tasks suggests that Area X and LMAN are presumably specialized brain regions implicated specifically in imitative vocal but not perceptual or other types of learning and motor activities that are linked to IEG expression. This conclusion is in agreement with three previous observations: (i) when adult male canaries learned to associate species-specific song with shock, no learning-induced ZENK expression was found in the Area X and LMAN [74], (ii) adult male zebra finches can learn to associate different song stimuli with different reward/punishment outcomes in the absence of Area X [75] and (iii) during different motor patterns IEGs were expressed only in areas adjacent to these nuclei but not within them [16].

Summing up, we have found that the structures of the song system can be divided into three categories in respect to their IEG response after an aversive food learning paradigm: 1) LMAN and Area X, which showed no transcriptional activation during the non-vocal learning task; 2) NCM, where induction of c-Fos and ZENK was observed after non-vocal behavior as well, but with less ZENK expression than induced by hearing song; 3) HVC and RA, in which ZENK expression was induced only by singing, whereas c-Fos expression was induced to an equal extent after the food aversion learning task and after singing.

### Relation of the brain regions involved in food discrimination learning in the zebra finch to the functional areas of the chicken brain

To search for explanations for the transcriptional activation in the songbird brain areas during food aversion learning we took into account known homologies in the avian brain, and we related those areas of zebra finches to the brain regions involved in food aversion learning in chickens. We therefore also examined brain areas of zebra finches that are homologous to the regions critical for long-term memory formation in passive avoidance learning in day old domestic chickens, namely IMM and MSt [31], [34], [76].

In IMM, patterns of expression for both c-Fos and ZENK in the food aversion group were similar to those in the active control and song control groups, so that it would be difficult to assess a contribution of learning to the induction of expression. IMM in songbirds could receive polysensory inputs from Ndc, as in chickens [35], [77]–[79], and processing of this information could require IEGs during foraging behavior, singing/song perception and food aversion learning alike.

Both IEGs were induced in MSt in the song, food aversion and active control groups, suggesting high level of plasticity-related processes during these types of behavior in this structure. Nevertheless, ZENK expression was significantly more elevated in the food aversion group relatively to the active control group. It is not clear, whether this was related to the learning itself, or to their agitated state after trying the aversant (e.g. additional flying could induce more ZENK expression as was reported for the anterior striatum of birds [16], though unlike in our experiments, in that study c-Fos expression was affected as well).

Summing up, transcriptional activation during food aversion learning in zebra finches appeared in similar areas as in chickens (IMM, Ndc and MSt).

### Comparison of learning-induced expression patterns of two immediate-early genes, ZENK and c-Fos, in the forebrain of zebra finch

Expression of the inducible transcription factors c-Fos and ZENK is considered to be a general marker of long-term synaptic changes required for long-term memory formation [36]–[40], [80]. However, patterns of c-Fos and ZENK expression have been rarely compared in the same brains, even though it is known that they can differ strikingly [24].

Curiously, IMM appeared to be the only analyzed structure where c-Fos and ZENK were expressed mainly in different neuronal populations, although the portions of neurons expressing each of these genes were similar, whereas in all other analyzed brain areas, when both genes were expressed this tended to occur in the same cells.

Nevertheless, c-Fos and ZENK had overall different patterns of expression. Most strikingly in the song system, ZENK expression seemed to be specific to the singing, while c-Fos was also expressed in other contexts. These differences probably reflect differential regulation by neural activity, for example through adrenergic receptors. Locus coeruleus (LoC) provides noradrenergic innervation to HVC [81], [82], and there are certain differences between the responses of ZENK and c-Fos gene expression to the input through adrenoceptors: For example, administration of N-(2-chloroethyl)-N-ethyl-2-bromobenzylamine, a selective noradrenergic neurotoxin, suppresses the basal and light-induced expression of c-Fos in the visual cortex [83], induces it in the hippocampus in adult rats [84] but has no effect on ZENK expression in the same animals. Thus, noradrenergic innervation could also regulate c-Fos expression in HVC without induction of ZENK. Activation of either of these genes alone or in combination potentially can lead to different modifications of neuronal circuits, because due to the different DNA-binding domains of the transcription factors coded by ZENK and c-Fos genes (zinc-fingers and leucine zippers, respectively [80], [85]), they can regulate expression of different late genes, performing different functions in neuronal plasticity. Thus, our work is in line with previous reports on differential IEG expression across brain regions linked to similar behaviors and underscores the need to use multiple IEGs to draw general conclusions.

In conclusion, our data suggest that despite their anatomical and functional specialization, some of the songbird brain regions (e.g., HVC and possibly RA) may participate in circuits mediating other behaviors (such as feeding or associations related to food). However, despite this anatomical overlap, the molecular mechanisms of neuronal recruitment into different functional systems (at the level of inducible transcription factors) may differ for the two types of behavior. Since some of the brain areas involved in speech production in humans (e.g., Broca's area) are also involved in gesture recognition [65], [67]–[69] and arm movements [66]–[69] it is tempting to speculate that brain regions for both human speech and birdsong evolved as specialization of preexisting motor pathways [4], [15], [16], [86], [87], some of which still retaining some of their older functions.

## Materials and Methods

### Animals and training procedures

Twenty eight male zebra finches (*Taeniopygia guttata*) were obtained commercially at the age of four months or older, the age when their adult song has developed into the stable adult form and the song system is mature [6], [88]. All experimental protocols were performed in accordance with the “Guidance to works involving animals in experiments” and approved by the Ethics Committee of P.K. Anokhin Institute of Normal Physiology, the Russian Academy of Medical Sciences: Protocol Nr.1, 3.09.2005. The birds were housed at the institute of Normal Physiology at constant 25 °C room temperature and with a 12∶12 hr light/dark cycle. During an adaptation period lasting seven days, males (two per cage, measuring 50×30×40 cm) were provided with water and standard seed mixture *ad libitum*. Additionally, Chinese cabbage leaves were provided once a day, and birds consumed those readily. On day seven, the males were caged individually, with visual and auditory contact to each other. Water and food continued to be available *ad libitum* but cabbage leaves were not given on the day before the experiment.

Birds were divided into four groups. One was exposed to the aversive food association learning (hence called ‘food aversion group’). Three groups served as controls, see below. The one-trial food aversion task commenced on day nine (10 birds). The birds were presented with a novel type of greens (parsley sprig) coated with undiluted bitter-tasting methylanthranilate solution (MeA, Sigma), which is used as an aversive cue in the passive avoidance learning model in chicken hatchlings. All zebra finches pecked at the MeA coated parsley only once, displaying a distinct disgust reaction (head shaking and beak wiping); the parsley remained in the cage for 5 min. The birds were tested 90 min after the training, when protein-synthesis dependent long-term memory formation for this task in chicken chicks is known to be completed [89]. For the test, the zebra finches were presented during 5 min with parsley and another unfamiliar green (dill sprig); both greens were not coated with MeA. The birds' behavior was videotaped (Sony DCR-TRV230E) and analyzed for the following features: approaches to and retreats from the two different herbs, latency to the first approach, and number of pecks, beak wiping and head shaking. Immediately after the test, the birds were sacrificed by decapitation, their brains dissected and frozen over liquid nitrogen and then stored at −70 °C until sectioned.

To test for natural food preferences between parsley and dill, 21 adult male zebra finches from the breeding facility at the Max-Planck Institute for Molecular Genetics, Berlin, were presented simultaneously with these greens in their home cages for 3 min; and their behavior was recorded by the experimenter.

To control for other variables than the aversive food learning, one group was treated identical to the food aversion group, except for being exposed to the aversive stimulus. We call this group ‘active control’. In this group, six zebra finches were given a Chinese cabbage leaf for 5 min on day nine; therefore, no aversive learning occurred in this group. All the birds pecked and ate from the leaves during the session. 90 min later the procedure was repeated, paralleling the test in the food aversion group, and immediately afterwards the birds were sacrificed and their brains frozen for sectioning.

The birds of the food aversion and active control groups did not sing during the hour before the experiment and during the experiments. Attempts of some of them to sing were prevented by the experimenter sitting next to the cage and raising a hand as soon as they sat in a characteristic pose and produced introductory notes.

To monitor the amount of gene expression induced in song motor nuclei associated with singing behavior [16], [28]–[30] and induced in auditory song nuclei as a result of hearing song [16], [27], [45], [90]–[92], a second control group (6 birds, called ‘song control group’) were presented simultaneously for 30 min daily during the adaptation period with recorded songs of two unfamiliar male zebra finches. This was intended to habituate the birds to this procedure and to habituate auditory song processing regions to those particular stimuli [92]. On the ninth day of the experiment, at the beginning of the light period, the birds were presented with a tape-recording of an adult male zebra finch song that they had not heard previously; the 2 sec long song segment was repeated twice per minute during a 15 min period. The frequency and duration of stimulation were shown to produce a robust IEG expression in NCM [90]. Playback of another male's song increases the likelihood of singing [29], [30], and those birds that did not sing spontaneously started singing within the first 10 min of the playback. The birds' behavior was registered by the experimenter and video recorded. 90 min after the start of the song presentation, i.e. at least 80 min after the birds had started to sing, they were killed, their brains frozen and stored until processed.

The third control group (‘quiet control’) consisted of six zebra finches that were kept in a dark, quiet room for 8 hrs during the night. Under these conditions, minimal IEG activity occurred [16], [28]. After this period the birds were decapitated and their brains processed as above.

The experiments were conducted in two series: during the first, we compared IEG expression visualized by immunoperoxidase (DAB) staining after food aversion learning with that in active and quiet control conditions, while in the second part we compared IEG expression visualized by immunofluorescent staining after food aversion learning with expression following auditory exposure to song and singing (song control).

### Brain sectioning, c-Fos and ZENK immunohistochemistry

20 µm transverse brain sections were cut on a cryostat at −18 °C. Sixteen sections per brain were thaw-mounted on glass slides and left to dry overnight at room temperature. The sections were then fixed in 4% paraformaldehyde at 4 °C and washed in phosphate buffer (PBS, pH 7.4).

#### DAB staining

The sections were treated with 0.3% H_2_0_2_ for 30 min, rinsed in PBS and incubated with 3% normal goat serum for 30 min. Incubation with primary antibodies against c-Fos (K-25, rabbit polyclonal IgG; 1∶1000; Santa Cruz Biotechnology) or ZENK (Egr-1, C-19, rabbit polyclonal IgG; 1∶600; Santa Cruz Biotechnology) was carried out overnight at room temperature. On the next day the sections were washed in PBS containing 0.3% of Triton X100 and then incubated for 2 hrs with secondary antibodies (anti-rabbit IgG; dilution 1∶200; Vector Laboratories). After washing in PBS/TritonX100 the slides were incubated for 1 hr in ABC (avidin/biotinylated enzyme complex; Elite ABC Kit, Vector Labs) and stained with diaminobenzidine. After dehydration in graded ethanols the sections were mounted using xylene-based mounting medium and coverslipped.

#### Fluorescent staining

Sections were incubated in 3% normal donkey serum for 30 min and then with primary antibodies overnight at room temperature. Antibodies against c-Fos and ZENK (see above) and NeuN (NeuN mouse monoclonal IgG; 1∶200; Chemicon) were applied simultaneously. After PBS/TritonX100 washing, the sections were incubated in the dark for 2 hrs with fluorescent secondary antibodies (Alexa Fluor 594 donkey anti-rabbit IgG, Alexa Fluor 488 donkey anti-goat IgG) and biotinylated horse antimouse IgG (Vector Labs), all dilutions 1∶400; and then with AMCA/AvidinD (Vector Labs) (1∶200) for 1 hr; rinsed and coverslipped with fluorescent mounting medium (DAKO).

These primary antibodies have been already used in zebra finches to identify c-Fos and ZENK expression in hippocampus during spatial learning [93]. To verify that the secondary antibodies did not produce non-specific staining, prior to experimental stainings, we performed negative control stainings omitting the primary antibodies, on brain tissue samples from the same animals.

### Image and data analysis

Briefly, the immunopositive cell nuclei were counted on digitized images acquired on a Olympus BX50 microscope with 4x, 10x and 20x objective using AnalySIS™ 3.0 image analysis software. For each brain, 8 transverse sections containing both hemispheres were analyzed, and for each structure, 4 frames were counted. Boundaries of the analyzed structures served as counting frame boundaries; two structures that did not have clear boundaries, NCM and Ndc, were analyzed using square frames of 0.6 mm^2^. All structures were defined according to the Stereotaxic Canary Brain Atlas [53], Stereotaxic Chicken Brain Atlas [94] and according to [95]. The structures analyzed in the current work and their localization within the brain are schematically depicted in **Figure 1**.

The data from birds from different groups were compared only within the set of experiments using the same type of antibodies: e.g., **Figure 2** shows examples of DAB staining of IEG expression in the birds from food aversion group, and **Figure 4 A**,**B** shows statistical comparison of the data from these birds with similarly processed active and quite controls; **Figure 3** shows examples of IEG expression in birds from the food aversion (left) and song control (right) groups that was revealed with fluorescent immunocytochemistry, and data from these birds were used for statistical analysis shown in **Figure 4C**–**F**. On the fluorescent images we set up the following color scheme to identify immunoreactive nuclei: blue for postmitotic NeuN-positive neurons, red for c-Fos-positive nuclei, and green for ZENK-positive nuclei (**Figure 6**). This allowed us to calculate the fraction of neurons expressing c-Fos or/and ZENK in relation to all neurons of each structure. Co-localization was also analyzed in 6 animals and 6 sections using confocal microscopy (**Figure S1**). To quantify immunoreactive nuclei automatically, we used the particle analysis feature of AnalySIS™ 3.0 software first setting corresponding color intensity thresholds and area size and shape of individual particles – immunopositive nuclei – and then applying the settings to the whole region of interest (song nuclei etc.). Automated particle analysis was validated by an experimenter checking randomly chosen immunopositive cells that were counted or miscounted by the software.

The differences in numbers and fractions of immunoreactive cells between groups were analyzed using one-way ANOVA followed by LSD post-hoc test in the first set of experiments (comparison of the food aversion group and active and quiet control groups) and independent-samples t-test in the second part (comparison between the food aversion and song control groups); for assessment of the interhemispheric differences within each group, paired-samples t-test was applied (SPSS 16.0). Correlations between the proportions of IEG expressing neurons and number of song bouts during the experiment (transcribed manually from recordings) were calculated using correlation matrices in Statistica© 6.0.

## Supporting Information

Figure S1 **A representative confocal image of fluorescent antibody staining revealing IEG expression in HVC after undirected singing.** HVC neurons expressed ZENK (green nuclear stain), c-Fos (red nuclear stain) or both genes simultaneously (yellow). Transverse section. Scale bar, 70 µm.
(TIF)

## References

[1] Bolhuis JJ, Okanoya K, Scharff C (2010). Twitter evolution: converging mechanisms in birdsong and human speech.. Nat Rev Neurosci.

[2] Fisher SE, Scharff C (2009). FOXP2 as a molecular window into speech and language.. Trends Genet.

[3] White SA (2009). Genes and vocal learning.. Brain Lang.

[4] Jarvis ED (2004). Learned birdsong and the neurobiology of human language.. Ann N Y Acad Sci.

[5] Phan ML, Vicario DS (2003). Hemispheric differences in processing of vocalizations depend on early experience.. Proc Natl Acad Sci U S A.

[6] Doupe AJ, Kuhl PK (1999). Birdsong and human speech: common themes and mechanisms.. Annu Rev Neurosci.

[7] Phan ML, Pytte CL, Vicario DS (2006). Early auditory experience generates long-lasting memories that may subserve vocal learning in songbirds.. Proc Natl Acad Sci U S A.

[8] Gahr M (2000). Neural song control system of hummingbirds: comparison to swifts, vocal learning (Songbirds) and nonlearning (Suboscines) passerines, and vocal learning (Budgerigars) and nonlearning (Dove, owl, gull, quail, chicken) nonpasserines.. J Comp Neurol.

[9] Simpson HB, Vicario DS (1990). Brain pathways for learned and unlearned vocalizations differ in zebra finches.. J Neurosci.

[10] Vicario DS, Simpson HB (1995). Electrical stimulation in forebrain nuclei elicits learned vocal patterns in songbirds.. J Neurophysiol.

[11] Jürgens U (2009). The neural control of vocalization in mammals: a review.. J Voice.

[12] Nishikawa KC (2002). Evolutionary convergence in nervous systems: insights from comparative phylogenetic studies.. Brain Behav Evol.

[13] Carr CE, Soares D (2002). Evolutionary convergence and shared computational principles in the auditory system.. Brain Behav Evol.

[14] Carr CE, Soares D, Smolders J, Simon JZ (2009). Detection of interaural time differences in the alligator.. J Neurosci.

[15] Farries MA (2001). The oscine song system considered in the context of the avian brain: lessons learned from comparative neurobiology.. Brain Behav Evol.

[16] Feenders G, Liedvogel M, Rivas M, Zapka M, Horita H (2008). Molecular mapping of movement-associated areas in the avian brain: a motor theory for vocal learning origin.. PLoS One.

[17] Margoliash D, Fortune ES, Sutter ML, Yu AC, Wren-Hardin BD (1994). Distributed representation in the song system of oscines: evolutionary implications and functional consequences.. Brain Behav Evol.

[18] Mello CV, Vates GE, Okuhata S, Nottebohm F (1998). Descending auditory pathways in the adult male zebra finch (Taeniopygia guttata).. J Comp Neurol.

[19] Watanabe S, Bischof HJ (2004). Effects of hippocampal lesions on acquisition and retention of spatial learning in zebra finches.. Behav Brain Res.

[20] Mayer U, Watanabe S, Bischof HJ (2009). Hippocampal activation of immediate early genes Zenk and c-Fos in zebra finches (Taeniopygia guttata) during learning and recall of a spatial memory task.. Neurobiol Learn Mem.

[21] Bailey DJ, Wade J, Saldanha CJ (2009). Hippocampal lesions impair spatial memory performance, but not song–a developmental study of independent memory systems in the zebra finch.. Dev Neurobiol.

[22] Bischof HJ, Rollenhagen A (1999). Behavioural and neurophysiological aspects of sexual imprinting in zebra finches.. Behav Brain Res.

[23] Lieshoff C, Grosse-Ophoff J, Bischof HJ (2004). Sexual imprinting leads to lateralized and non-lateralized expression of the immediate early gene zenk in the zebra finch brain.. Behav Brain Res.

[24] Bailey DJ, Wade J (2003). Differential expression of the immediate early genes FOS and ZENK following auditory stimulation in the juvenile male and female zebra finch.. Brain Res Mol Brain Res.

[25] Bailey DJ, Wade J (2005). FOS and ZENK responses in 45-day-old zebra finches vary with auditory stimulus and brain region, but not sex.. Behav Brain Res.

[26] Rose SP (2000). God's organism? The chick as a model system for memory studies.. Learn Mem.

[27] Mello CV, Vicario DS, Clayton DF (1992). Song presentation induces gene expression in the songbird forebrain.. Proc Natl Acad Sci U S A.

[28] Jarvis ED, Nottebohm F (1997). Motor-driven gene expression.. Proc Natl Acad Sci U S A.

[29] Jarvis ED, Scharff C, Grossman MR, Ramos JA, Nottebohm F (1998). For whom the bird sings: context-dependent gene expression.. Neuron.

[30] Kimpo RR, Doupe AJ (1997). FOS is induced by singing in distinct neuronal populations in a motor network.. Neuron.

[31] Kossut M, Rose SP (1984). Differential 2-deoxyglucose uptake into chick brain structures during passive avoidance training.. Neuroscience.

[32] Davies DC, Taylor DA, Johnson MH (1988). The effects of hyperstriatal lesions on one-trial passive-avoidance learning in the chick.. J Neurosci.

[33] Patterson TA, Gilbert DB, Rose SP (1990). Pre- and post-training lesions of the intermediate medial hyperstriatum ventrale and passive avoidance learning in the chick.. Exp Brain Res.

[34] Anokhin KV, Mileusnic R, Shamakina IY, Rose SP (1991). Effects of early experience on c-fos gene expression in the chick forebrain.. Brain Res.

[35] Metzger M, Jiang S, Braun K (1998). Organization of the dorsocaudal neostriatal complex: a retrograde and anterograde tracing study in the domestic chick with special emphasis on pathways relevant to imprinting.. J Comp Neurol.

[36] Clayton DF (1997). Role of gene regulation in song circuit development and song learning.. J Neurobiol.

[37] Guzowski JF (2002). Insights into immediate-early gene function in hippocampal memory consolidation using antisense oligonucleotide and fluorescent imaging approaches.. Hippocampus.

[38] Poirier R, Cheval H, Mailhes C, Garel S, Charnay P (2008). Distinct functions of egr gene family members in cognitive processes.. Front Neurosci.

[39] Alberini CM (2009). Transcription factors in long-term memory and synaptic plasticity.. Physiol Rev.

[40] Mileusnic R, Anokhin K, Rose SP (1996). Antisense oligodeoxynucleotides to c-fos are amnestic for passive avoidance in the chick.. Neuroreport.

[41] London SE, Clayton DF (2008). Functional identification of sensory mechanisms required for developmental song learning.. Nat Neurosci.

[42] Kabelik D, Kelly AM, Goodson JL (2010). Dopaminergic regulation of mate competition aggression and aromatase-Fos colocalization in vasotocin neurons.. Neuropharmacology.

[43] Svec LA, Licht KM, Wade J (2009). Pair bonding in the female zebra finch: a potential role for the nucleus taeniae.. Neuroscience.

[44] Velho TA, Mello CV (2008). Synapsins are late activity-induced genes regulated by birdsong.. J Neurosci.

[45] Velho TA, Pinaud R, Rodrigues PV, Mello CV (2005). Co-induction of activity-dependent genes in songbirds.. Eur J Neurosci.

[46] Mason JR, Epple G (1998). Evaluation of bird repellent additives to a simulated pesticide carrier formation.. Crop Protection.

[47] Bull R, Ferrera E, Orrego F (1976). Effects of anisomycin on brain protein synthesis and passive avoidance learning in newborn chicks.. J Neurobiol.

[48] Vicario DS (2004). Using learned calls to study sensory-motor integration in songbirds.. Ann N Y Acad Sci.

[49] Wada K, Howard JT, McConnell P, Whitney O, Lints T (2006). A molecular neuroethological approach for identifying and characterizing a cascade of behaviorally regulated genes.. Proc Natl Acad Sci U S A.

[50] Gobes SM, Ter Haar SM, Vignal C, Vergne AL, Mathevon N (2009). Differential responsiveness in brain and behavior to sexually dimorphic long calls in male and female zebra finches.. J Comp Neurol.

[51] Phillmore LS, Bloomfield LL, Weisman RG (2003). Effects of songs and calls on ZENK expression in the auditory telencephalon of field- and isolate-reared black capped chickadees.. Behav Brain Res.

[52] Long KD, Kennedy G, Salbaum JM, Balaban E (2002). Auditory stimulus-induced changes in immediate-early gene expression related to an inborn perceptual predisposition.. J Comp Physiol A Neuroethol Sens Neural Behav Physiol.

[53] Stokes TM, Leonard CM, Nottebohm F (1974). The telencephalon, diencephalon, and mesencephalon of the canary, Serinus canaria, in stereotaxic coordinates.. J Comp Neurol.

[54] Zeigler HP, Marler P, Zeigler HP, Marler P (2008). Neuroscience of Birdsong, ed..

[55] Vicario DS (1993). A new brain stem pathway for vocal control in the zebra finch song system.. Neuroreport.

[56] Wild JM, Kubke MF, Mooney R (2009). Avian nucleus retroambigualis: cell types and projections to other respiratory-vocal nuclei in the brain of the zebra finch (Taeniopygia guttata).. J Comp Neurol.

[57] Wild JM (2004). Functional neuroanatomy of the sensorimotor control of singing.. Ann N Y Acad Sci.

[58] Vicario DS (1991). Contributions of syringeal muscles to respiration and vocalization in the zebra finch.. J Neurobiol.

[59] Shank SS, Margoliash D (2009). Sleep and sensorimotor integration during early vocal learning in a songbird.. Nature.

[60] Mistry S, Hamdy S (2008). Neural control of feeding and swallowing.. Phys Med Rehabil Clin N Am.

[61] Sawczuk A, Mosier KM (2001). Neural control of tongue movement with respect to respiration and swallowing.. Crit Rev Oral Biol Med.

[62] Hoese WJ, Podos J, Boetticher NC, Nowicki S (2000). Vocal tract function in birdsong production: experimental manipulation of beak movements.. J Exp Biol.

[63] Ohms VR, Snelderwaard P, Ten Cate C, Beckers GJ (2010). Vocal tract articulation in zebra finches.. PLoS One.

[64] Dubbeldam JL (1997). Intratelencephalic Sensorimotor Circuits in Birds - What Have Feeding and Vocalization in Common?. Netherlands Journal of Zoology.

[65] Fadiga L, Craighero L, Destro MF, Finos L, Cotillon-Williams N (2006). Language in shadow.. Soc Neurosci.

[66] Gentilucci M, Dalla Volta R (2008). Spoken language and arm gestures are controlled by the same motor control system.. Q J Exp Psychol (Colchester).

[67] Rizzolatti G, Arbib MA (1998). Language within our grasp.. Trends Neurosci.

[68] Arbib MA (2006). A sentence is to speech as what is to action?. Cortex.

[69] Arbib MA (2008). From grasp to language: embodied concepts and the challenge of abstraction.. J Physiol Paris.

[70] Prather JF, Peters S, Nowicki S, Mooney R (2008). Precise auditory-vocal mirroring in neurons for learned vocal communication.. Nature.

[71] Aronov D, Andalman AS, Fee MS (2008). A specialized forebrain circuit for vocal babbling in the juvenile songbird.. Science.

[72] Jin H, Clayton DF (1997). Localized changes in immediate-early gene regulation during sensory and motor learning in zebra finches.. Neuron.

[73] Stripling R, Volman SF, Clayton DF (1997). Response modulation in the zebra finch neostriatum: relationship to nuclear gene regulation.. J Neurosci.

[74] Jarvis ED, Mello CV, Nottebohm F (1995). Associative learning and stimulus novelty influence the song-induced expression of an immediate early gene in the canary forebrain.. Learn Mem.

[75] Scharff C, Nottebohm F, Cynx J (1998). Conspecific and heterospecific song discrimination in male zebra finches with lesions in the anterior forebrain pathway.. J Neurobiol.

[76] Patterson TA, Rose SP (1992). Memory in the chick: multiple cues, distinct brain locations.. Behav Neurosci.

[77] Metzger M, Jiang S, Wang J, Braun K (1996). Organization of the dopaminergic innervation of forebrain areas relevant to learning: a combined immunohistochemical/retrograde tracing study in the domestic chick.. J Comp Neurol.

[78] Braun K, Bock J, Metzger M, Jiang S, Schnabel R (1999). The dorsocaudal neostriatum of the domestic chick: a structure serving higher associative functions.. Behav Brain Res.

[79] Bock J, Thode C, Hannemann O, Braun K, Darlison MG (2005). Early socio-emotional experience induces expression of the immediate-early gene Arc/arg3.1 (activity-regulated cytoskeleton-associated protein/activity-regulated gene) in learning-relevant brain regions of the newborn chick.. Neuroscience.

[80] Herdegen T, Leah JD (1998). Inducible and constitutive transcription factors in the mammalian nervous system: control of gene expression by Jun, Fos and Krox, and CREB/ATF proteins.. Brain Res Brain Res Rev.

[81] Mello CV, Pinaud R, Ribeiro S (1998). Noradrenergic system of the zebra finch brain: immunocytochemical study of dopamine-beta-hydroxylase.. J Comp Neurol.

[82] Hara E, Kubikova L, Hessler NA, Jarvis ED (2007). Role of the midbrain dopaminergic system in modulation of vocal brain activation by social context.. Eur J Neurosci.

[83] Yamada Y, Hada Y, Imamura K, Mataga N, Watanabe Y (1999). Differential expression of immediate-early genes, c-fos and zif268, in the visual cortex of young rats: effects of a noradrenergic neurotoxin on their expression.. Neuroscience.

[84] Sanders JD, Happe HK, Bylund DB, Murrin LC (2008). Differential effects of neonatal norepinephrine lesions on immediate early gene expression in developing and adult rat brain.. Neuroscience.

[85] O'Donovan KJ, Tourtellotte WG, Millbrandt J, Baraban JM (1999). The EGR family of transcription-regulatory factors: progress at the interface of molecular and systems neuroscience.. Trends Neurosci.

[86] Lieberman P (2002). On the nature and evolution of the neural bases of human language.. Am J Phys Anthropol.

[87] Dubbeldam JL (1998). The neural substrate for ‘learned’ and ‘nonlearned’ activities in birds: a discussion of the organization of bulbar reticular premotor systems with side-lights on the mammalian situation.. Acta Anat (Basel).

[88] Immelman K (1969). Song development in the zebra finch and other estrilid finches, in Hinde RA, Editor Bird vocalizations..

[89] Gibbs ME, Ng KT (1979). Behavioural stages in memory formation.. Neurosci Lett.

[90] Kruse AA, Stripling R, Clayton DF (2000). Minimal experience required for immediate-early gene induction in zebra finch neostriatum.. Neurobiol Learn Mem.

[91] Kruse AA, Stripling R, Clayton DF (2004). Context-specific habituation of the zenk gene response to song in adult zebra finches.. Neurobiol Learn Mem.

[92] Mello C, Nottebohm F, Clayton D (1995). Repeated exposure to one song leads to a rapid and persistent decline in an immediate early gene's response to that song in zebra finch telencephalon.. J Neurosci.

[93] Mayer U, Watanabe S, Bischof HJ (2009). Hippocampal activation of immediate early genes Zenk and c-Fos in zebra finches (Taeniopygia guttata) during learning and recall of a spatial memory task.. Neurobiol Learn Mem.

[94] Kuenzel WJ, Masson M (1988). A Stereotaxic Atlas of the brain of the chick..

[95] Wild JM, Farabaugh SM (1996). Organization of afferent and efferent projections of the nucleus basalis prosencephali in a passerine, Taeniopygia guttata.. J Comp Neurol.

