# Supplementary figures and images for: Food for Song: Expression of C-Fos and ZENK in the Zebra Finch Song Nuclei during Food Aversion Learning
Source: PLoS One. 2011 Jun 10;6(6):e21157. doi: 10.1371/journal.pone.0021157 (PMC3112232; doi:10.1371/journal.pone.0021157)

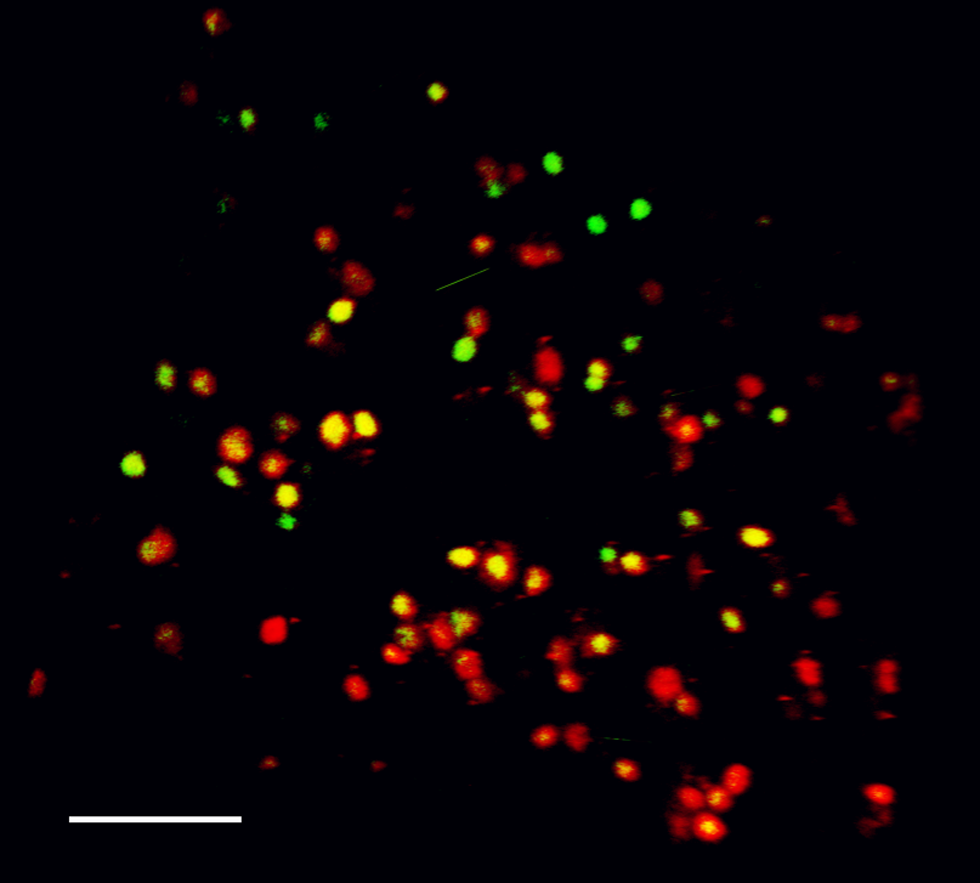

Supplement: Figure S1 — A representative confocal image of fluorescent antibody staining revealing IEG expression in HVC after undirected singing. HVC neurons expressed ZENK (green nuclear stain), c-Fos (red nuclear stain) or both genes simultaneously (yellow). Transverse section. Scale bar, 70 µm. (TIF) [file pone.0021157.s001.tif]
